# Tolerating Factor VIII: Recent Progress

**DOI:** 10.3389/fimmu.2019.02991

**Published:** 2020-01-10

**Authors:** Sebastien Lacroix-Desmazes, Jan Voorberg, David Lillicrap, David W. Scott, Kathleen P. Pratt

**Affiliations:** ^1^Centre de Recherche des Cordeliers, INSERM, Sorbonne Université, Université de Paris, Paris, France; ^2^Sanquin Research and Landsteiner Laboratory, Department of Molecular and Cellular Hemostasis, Academic Medical Center, University of Amsterdam, Amsterdam, Netherlands; ^3^Department of Pathology and Molecular Medicine, Queen's University, Kingston, ON, Canada; ^4^Uniformed Services University of the Health Sciences, Bethesda, MD, United States

**Keywords:** factor VIII, protein immunogenicity, hemophilia A, peripheral tolerance, immune tolerance induction, antigen presentation, T-cell engineering

## Abstract

Development of neutralizing antibodies against biotherapeutic agents administered to prevent or treat various clinical conditions is a longstanding and growing problem faced by patients, medical providers and pharmaceutical companies. The hemophilia A community has deep experience with attempting to manage such deleterious immune responses, as the lifesaving protein drug factor VIII (FVIII) has been in use for decades. Hemophilia A is a bleeding disorder caused by genetic mutations that result in absent or dysfunctional FVIII. Prophylactic treatment consists of regular intravenous FVIII infusions. Unfortunately, 1/4 to 1/3 of patients develop neutralizing anti-FVIII antibodies, referred to clinically as “inhibitors,” which result in a serious bleeding diathesis. Until recently, the only therapeutic option for these patients was “Immune Tolerance Induction,” consisting of intensive FVIII administration, which is extraordinarily expensive and fails in ~30% of cases. There has been tremendous recent progress in developing novel potential clinical alternatives for the treatment of hemophilia A, ranging from encouraging results of gene therapy trials, to use of other hemostatic agents (either promoting coagulation or slowing down anti-coagulant or fibrinolytic pathways) to “bypass” the need for FVIII or supplement FVIII replacement therapy. Although these approaches are promising, there is widespread agreement that preventing or reversing inhibitors remains a high priority. Risk profiles of novel therapies are still unknown or incomplete, and FVIII will likely continue to be considered the optimal hemostatic agent to support surgery and manage trauma, or to combine with other therapies. We describe here recent exciting studies, most still pre-clinical, that address FVIII immunogenicity and suggest novel interventions to prevent or reverse inhibitor development. Studies of FVIII uptake, processing and presentation on antigen-presenting cells, epitope mapping, and the roles of complement, heme, von Willebrand factor, glycans, and the microbiome in FVIII immunogenicity are elucidating mechanisms of primary and secondary immune responses and suggesting additional novel targets. Promising tolerogenic therapies include development of FVIII-Fc fusion proteins, nanoparticle-based therapies, oral tolerance, and engineering of regulatory or cytotoxic T cells to render them FVIII-specific. Importantly, these studies are highly applicable to other scenarios where establishing immune tolerance to a defined antigen is a clinical priority.

## Introduction

Factor VIII (FVIII) is an essential blood coagulation cofactor. Recombinant or plasma-derived FVIII is a lifesaving protein drug for hemophilia A (HA) patients, whose *F8* gene mutations result in either a complete lack of endogenous FVIII or in a circulating dysfunctional FVIII. Unfortunately, immune responses to FVIII resulting in neutralizing anti-FVIII antibodies, or “inhibitors,” complicate or preclude effective FVIII replacement therapy in a substantial fraction of HA patients. Inhibitors typically develop early in the course of FVIII replacement therapy, with a peak incidence occurring within the first 10–15 exposure days (1, 2). Longer-term surveillance studies indicate, however, that a substantial fraction of inhibitors develop after age 5, and that incidences increase again after age 50 (3). Inhibitor development in non-HA individuals also occurs as a rare but serious autoimmune reaction that is typically diagnosed subsequent to unexplained bleeding (4), primarily in the elderly, or following trauma, surgery or childbirth. Both allo- and autoimmune FVIII-specific antibodies are class-switched, as is typical for CD4^+^ T-cell driven immune responses (5, 6).

This review focuses on mechanisms of factor VIII immunogenicity and novel approaches to promote immune tolerance to this important protein drug. Despite decades of clinical experience with both plasma-derived and recombinant (r)FVIII products, there is still much to be learned about risk factors for inhibitor development and mechanisms of the anti-FVIII immune response. It is hoped that improved mechanistic understanding will lead to identification of reliable prognostic biomarkers and, even more significantly, of novel targets to promote immune tolerance to FVIII. An ideal therapeutic intervention would tolerize the individual specifically to FVIII, thereby avoiding the potential side effects of general immunosuppression. We focus on recent advances, some of which are being tested in current clinical trials, and others that have the potential for future clinical translation, e.g., animal model studies and *in vitro* experiments utilizing donated human blood samples.

The armamentarium available to treat HA patients has expanded significantly over the past decade. It currently includes rFVIII products produced in mammalian cell culture systems and rFVIII proteins that have been engineered to create sequence-modified or fusion proteins, or covalently modified, e.g., by PEGylation to extend their half-life. In addition, non-FVIII therapies that either mimic FVIII cofactor activity, or that target specific pro-coagulant or anti-coagulant pathways by shifting hemostasis to a more pro-coagulant phenotype and thereby prevent hemophilic bleeds, are now available, in preclinical testing, and in clinical trials. Three recently introduced non-FVIII options to treat HA are the bispecific antibody emicizumab (Hemlibra) (7, 8), the anti-Tissue Factor Pathway Inhibitor (TFPI) monoclonal antibody concizumab (9) and an RNAi targeting antithrombin (Fitusiran). These products, and several others that are in various stages in the translational pipeline, are described in more detail below. They present patients with non-FVIII options; this is particularly important for those who have developed inhibitors that preclude effective prevention or treatment of bleeds with FVIII. Some also show promise as therapeutics for hemophilia B (lack of functional factor IX) and other bleeding disorders. Because of its earlier introduction and approval, there is more clinical experience with emicizumab, which a growing number of patients are choosing for prophylactic management of HA. All of these therapies offer significant benefits in terms of convenience, as they do not involve the frequent intravenous infusions required for FVIII prophylaxis. Importantly, however, they cannot induce tolerance to FVIII unless they are administered in formulations that include the FVIII antigen. Furthermore, there is still limited experience with their use, effectiveness, and risk profiles in settings of trauma and surgery, when FVIII supplementation may well be required to prevent or reverse breakthrough bleeds. Therefore, the induction and maintenance of immune tolerance to FVIII remains a vital issue for all HA patients, regardless of which therapeutic product they utilize for routine prophylaxis.

Animal model studies continue to be essential for understanding mechanisms of FVIII immunogenicity and peripheral tolerance, as well as for testing novel therapies to identify candidates for possible clinical translation (10). Most animal studies of anti-FVIII immune responses have utilized HA mice with a targeted disruption of the *F8* gene, due to their lower cost and the greater availability of appropriate reagents and well-defined genetic strains, compared to larger animal models. However, large animal models have provided essential models of hemophilia A and B, especially for preclinical testing of various therapies. Gene therapy studies have relied for years on the use of HA dog colonies (11, 12). Interestingly, successful delivery of an *F8* transgene has not only corrected the HA bleeding phenotype, in some cases for years, but it has also shown promise as a potential therapy to achieve peripheral tolerance to FVIII (13). Human *F8* gene therapy trials have so far enrolled only adults with no prior FVIII inhibitor, so the possibility that gene therapy may have a tolerogenic effect on the naïve human immune response to FVIII remains untested.

Clinical Immune Tolerance Induction (ITI) therapy currently consists of intensive intravenous FVIII administration, which is challenging for patients/families, extraordinarily expensive, and fails in 25–30% of patients (14, 15). Unfortunately, attempts to “tolerize” HA mice via intensive FVIII infusions, analogous to clinical ITI protocols, have not yet been successful (16), although high-dose FVIII administration has been shown to suppress memory B cells *in vitro* (17). Therefore, further human studies are needed to identify biomarkers and potential new targets that could be manipulated to improve current clinical ITI success rates. Animal models allow studies of immune compartments in addition to the periphery, notably of the spleen and possibly the liver as major sites for the naïve response to intravenously administered FVIII. Recent advances in both animal and human studies of FVIII immunogenicity and tolerance are summarized below.

## Anti-FVIII Antibodies

Inhibitors are, by definition, neutralizing anti-FVIII antibodies, with titers reported in “Bethesda units” as measured by a clotting assay (18, 19). More comprehensive immunoprofiling efforts have incorporated measurements of total anti-FVIII antibody titers (expressed as dilution factors) and antibody isotypes/subclasses using ELISAs (20–22), surface plasmon resonance (23), and fluorescent bead-based assays (21, 24). FVIII-specific antibodies isolated from HA patients with an inhibitor response are primarily of subclasses IgG1 and IgG4 (25, 26), although lower levels of FVIII-specific IgG2 and IgG3 have also been detected and quantified in patients' plasma (23). Analysis of samples from 371 HA subjects (21% inhibitor-positive) showed a correlation of anti-FVIII IgG1, IgG2, and IgG4 with inhibitor development (27), indicating involvement of both Th1 and Th2 CD4^+^ T cells (5), while a separate study of 101 HA subjects (24% inhibitor-positive) and 19 autoimmune subjects revealed that neutralizing antibodies had higher apparent affinities for FVIII compared to non-neutralizing antibodies (22). Anaphylaxis is not a feature of anti-FVIII allo- or autoimmune responses.

A seminal 1992 study of 500 plasma samples from healthy, non-hemophilic donors revealed that an appreciable fraction contained anti-FVIII antibodies that were detectable by Bethesda and/or ELISA assays (28). A subsequent study reported isolation of anti-FVIII antibodies from non-hemophilic plasma, epitope mapping via competition ELISA assays, and characterization of anti-idiotypic antibodies that could block the interactions between FVIII and the natural anti-FVIII antibodies (29). In a more recent study, non-neutralizing anti-FVIII antibodies were detected in ~20% of plasma samples from >600 healthy non-HA blood donors (21, 30), although their predominant recognition of the heavily glycosylated B domain of FVIII indicated the binding of many of these “natural” antibodies may not have been strictly specific for FVIII. Anti-FVIII antibodies have also been quantified using a sensitive Luminex-based assay (24), which detected low-titer FVIII-binding antibodies in the vast majority of ~400 HA subjects, most of whom did not have a current inhibitor detectable by a clotting (Bethesda) assay. Interestingly, this study also detected low-titer FVIII-specific antibodies in an appreciable fraction of healthy non-HA control plasma samples, although titers were significantly lower (31).

## FVIII Uptake, Processing, and Presentation

**Figure 1:**
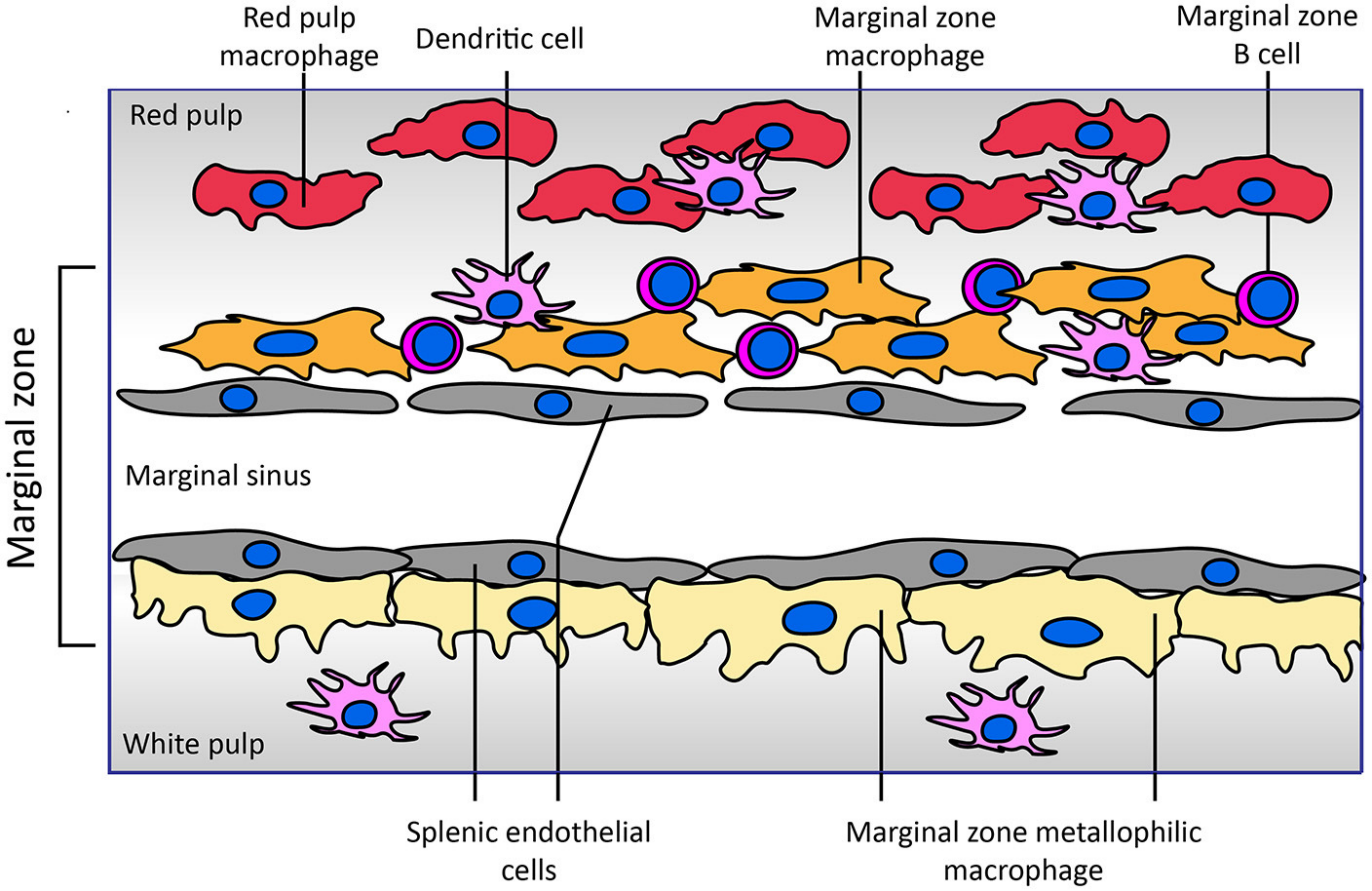
Architecture of the marginal zone of the mouse spleen. The image, adapted from Figure 4 of Mebius and Kraal (35), displays the cellular composition of the marginal zone of the spleen. Sampling of antigens from the circulating blood by different populations of antigen presenting cells surrounding the marginal sinus appears to be crucial for development of an adaptive immune response to FVIII. Both SIGNR1-positive marginal zone macrophages and CD169-positive marginal zone metallophilic macrophages have been implicated in the endocytosis of FVIII. Splenic endothelial cells lining the marginal sinus or red pulp sinus (not displayed in figure) expressing stabilin-2 may also contribute to FVIII internalization. Functional involvement of marginal zone B cells present within the marginal zone in the immune response has been inferred from depletion approaches. Different populations of dendritic cells reside in the spleen; the role of specific subsets of these cells in the development of FVIII inhibitors has not yet been explored. In contrast to that of the mouse spleen, the cellular architecture of the human spleen has not yet been fully elucidated (36, 37). In human spleen the border between the white and red pulp is formed by a perifollicular zone whose cellular composition has not yet been fully characterized.

The uptake of blood coagulation FVIII by antigen presenting cells has been studied intensively in the last decade. Most of these studies have been performed in model systems like human monocyte-derived dendritic cells as well as mouse bone marrow-derived dendritic cells (32–34). In parallel, immuno-localization of FVIII in the spleen following its infusion into mice has generated very interesting findings on the topology of FVIII association with, and possibly uptake by, different populations of antigen presenting cells in specific niches within the splenic architecture (Figure 1). The afferent small vessels in the spleen are lined by fenestrated endothelial cells that allow for interaction of circulating antigens with antigen presenting cells underlying this layer of endothelial cells.

A landmark study by Navarrete et al. provided compelling evidence for the association of FVIII with marginal zone metallophilic macrophages (38, 39). These results were confirmed in studies in which co-localization of infused FVIII with the marginal zone macrophages was observed based on its co-localization with macrophage marker proteins SIGNR1 and MARCO (35, 39). In addition, based on the co-localization of FVIII with SIGLEC-1, marginal zone macrophages located in the white pulp have also been implicated in processing of FVIII. The role of (metallophilic) marginal zone macrophages in *in vivo* uptake does not exclude that other populations of splenic cells also contribute to FVIII immunogenicity. Indeed, the depletion of splenic marginal zone B cells abrogated inhibitor development in a mouse model of HA (40). Marginal zone B cells are efficient scavengers for antigens that circulate in blood. Based on their localization in the spleen and their role in mounting an immune response, it is possible that FVIII is transported by them to different populations of antigen presenting cells following its retrieval from blood. However, recent Bruton's tyrosine kinase inhibition pre-clinical studies suggest no role for naïve B cells in development of a primary anti-FVIII immune response (41).

The architecture of the spleen promotes intimate contact between blood borne antigens and antigen capturing and presenting cells that are localized in the marginal zone (36, 37). Fenestrated splenic endothelial cells are well-positioned to filter blood borne antigens like FVIII and von Willebrand factor (VWF) from the circulating blood, thus promoting capture of antigens via scavenger receptors such as stabilin-2 (42). Marginal zone macrophages, marginal zone B cells and populations of marginal zone-located dendritic cells are also capable of capturing and/or processing blood borne antigens (36, 37). Transfer of antigens from marginal zone macrophages and endothelial cells to more dynamic marginal zone B cells and marginal zone-residing dendritic cells is most likely required for their transport to the T cell-enriched white pulp. Subsequent steps of FVIII transport are not yet well-defined, but antigen transfer to dendritic cells is required for primary immune responses, and FVIII presentation to naïve T cells and B cells is expected to occur primarily in the spleen (38). T follicular helper cells within germinal centers both select FVIII-specific B cells and drive affinity maturation and class-switching of their B-cell receptors, ultimately generating plasma cells that secrete high-affinity antibodies. Indeed, FVIII-deficient mice showed increased germinal center formation, proliferation of splenic T-follicular helper cells *in vitro*, and accumulation of T-follicular CD4^+^ T cells in the spleen following FVIII immunization (43).

Apart from the spleen, FVIII has also been shown to accumulate in macrophages in the liver (44). The precise population of antigen presenting cells in this organ have not yet been defined, but both liver sinusoidal endothelial cells and Kupffer cells have been shown to express endocytic receptors capable of internalizing FVIII and/or VWF (44). Both FVIII and VWF were shown to be endocytosed primarily by CD69^+^ Kupffer cells (44). More recently, liver sinusoidal endothelial cells have also been implicated in FVIII and VWF internalization (45). The liver is considered to provide a tolerogenic environment which supports the generation as well as proliferation of CD4^+^ T cells with a regulatory phenotype. LSEC are instrumental in the generation of regulatory CD4^+^ T cells, as they can efficiently endocytose and process blood borne antigens, thereby sequestering them in a relatively tolerogenic compartment, at least in the absence of significant inflammation (46). LSEC have been shown to express MHC class II, which can be further up-regulated upon inflammatory stimulation. Unlike dendritic cells, LSEC express limited amounts of co-stimulatory molecules and therefore are unable to direct the formation of classical CD4^+^ T helper cells (46). In apparent contrast with the tolerogenic role of LSEC, it was recently shown that stabilin-2 driven internalization of human FVIII/VWF complexes provides a crucial step in FVIII inhibitor development (45). Since stabilin-2 is expressed by both liver and splenic endothelial cells (42), uptake of FVIII-VWF complexes by these cells may also explain the modulating effects of stabilin-2 on FVIII immunogenicity.

**Figure 2:**
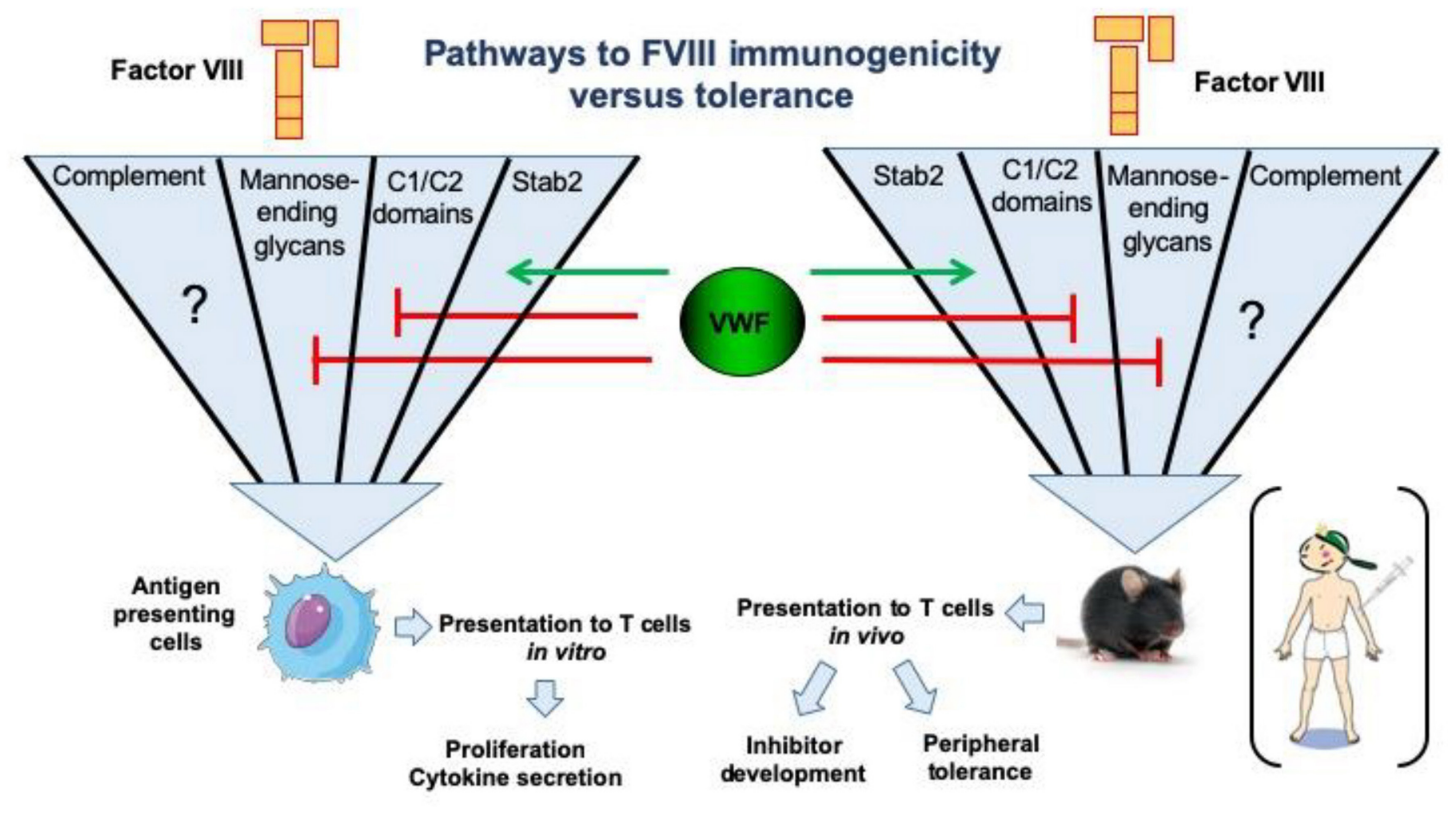
Pathways for endocytosis of FVIII by antigen-presenting cells and repercussions on FVIII immunogenicity or tolerance. The uptake of human FVIII has been studied *in vitro* using human antigen presenting cells, such as monocyte-derived dendritic cells. The major pathways identified to date include key positively charged amino-acids in the C1 and C2 domains of the FVIII molecule, as well as mannose-ending glycans at position N2118 in the C1 domain. Importantly, the later endocytic pathways are blocked in the presence of VWF. Conversely, activation of complement is able to restore the uptake of the FVIII C1 domain mutant Arg^2090^Ala, Lys^2092^Ala, and Met^2093^Ala. It is not known as yet whether complement activation overcomes the blocking effect of VWF in FVIII endocytosis. The stabilin-2 (Stab2)-dependent internalization pathway was demonstrated using Stab2-expressing HEK293 cells and also *in vivo* in mice. It is dependent on the presence of VWF. Interestingly, the neutralization of some endocytic pathways reduces the immunogenicity of FVIII in FVIII-deficient mice, particularly in the case of the Stab2- and complement C3b-dependent pathways (58).

Most of our knowledge of surface receptors implicated in FVIII endocytosis has been derived from *in vitro* studies. Different families of endocytic receptors have emerged during evolution to promote early processing of foreign antigens. Most of these receptors recognize distinct pattern-like structures which include glycans such as sialic acid (Siglec family of surface receptors), mannose or galactose structures on protein antigens (26, 47, 48). Additional classes of surface receptors recognizing more heterologous structures on protein antigens have also been implicated in FVIII endocytosis (32, 33, 47). The LDL-related low-density receptor 1 (LRP1) was identified as an endocytic receptor for FVIII (49, 50). The physiological importance of this receptor and other members of this receptor family is thought to be related primarily to FVIII clearance. Despite its abundance on antigen presenting cells, current evidence suggests that its role in FVIII presentation and immunogenicity is limited (51, 52). In contrast to LRP1, the mannose receptor has been firmly implicated in FVIII endocytosis by human dendritic cells (48). The mannose receptor is composed of a discrete series of repeated carbohydrate ligand-binding sites, one of which binds with high affinity to FVIII. This binding and endocytosis could be partially abrogated by mannan, and incubation of FVIII with dendritic cells in the presence of mannan completely inhibited proliferation of a FVIII-specific T-cell clone (48). Therefore, it was concluded that the mannose receptor is involved in immune recognition of FVIII by antigen presenting cells (48). Asn^239^ in the A1 domain of FVIII and Asn^2118^ in the C1 domain are attached to a glycan terminating in mannose, suggesting a mechanism for FVIII internalization by antigen presenting cells via the mannose receptor (53, 54). Complementary experiments employing murine bone marrow-derived dendritic cells, however, did not support a direct role for the mannose receptor in FVIII internalization (55). These findings suggest that, at least in mice, the mannose receptor may not be directly involved in FVIII immunogenicity; the extent to which FVIII immunogenicity in humans depends on mannose receptors remains to be established. Apart from the mannose-ending glycans linked to Asn^239^ and Asn^2118^, exposed surface loops in the C-terminal FVIII C1 and C2 domains containing positively-charged residues have been implicated in the uptake of FVIII by both human (monocyte-derived) and murine (bone marrow-derived) dendritic cells (52, 56, 57). Modification of residues in the C1 domain surface loop containing Arg^2090^, Lys^2092^, and Met^2093^ resulted in reduced FVIII inhibitor titers in FVIII-deficient mice (56). Interestingly, in one study the reduced immunogenicity was observed only in FVIII-VWF deficient mice, suggesting that the immunogenicity of this engineered FVIII variant was modulated by its binding to VWF (57). Likewise, modification of residues in the C2 domain surface loop containing Arg^2215^ and Arg^2220^ resulted in drastically reduced FVIII uptake by human dendritic cells (57). The roles of specific FVIII regions and receptors in binding, uptake by various cell types, antigenic processing and clearance of FVIII are the subject of ongoing research. An overview of our current understanding of FVIII uptake, processing and presentation on immunogenicity vs. tolerance is shown in Figure 2.

Since FVIII circulates in complex with its physiological, multimeric carrier protein VWF, it is not surprising that its endocytosis and potential immunogenicity are both modulated by VWF. *In vitro* studies have firmly established that in the presence of VWF the amount of FVIII that is internalized by antigen presenting cells is greatly reduced (59, 60). Following this internalization, FVIII is processed into peptides by endo-lysosomal proteases for subsequent loading onto MHC class II and presentation to CD4^+^ T cells (61). In agreement with its role in decreasing FVIII internalization, it has also been shown that VWF modulates the efficiency of FVIII peptide presentation (60, 62). These effects of VWF have been suggested as an underlying mechanism for the apparent reduced immunogenicity of VWF-containing (plasma-derived) FVIII concentrates, compared to rFVIII products, that was reported in the recent SIPPET study (2).

Studies in which monocyte-derived dendritic cells were pulsed with FVIII, followed by peptide elution and identification by LC-mass spectrometry, have yielded valuable insights into the repertoires of FVIII-derived peptides that can be presented on various MHC class II, and thereby made available for potential recognition by CD4^+^ T cells (60–65). Together with complementary studies characterizing CD4^+^ T-cell responses, which are discussed in detail later in this review, this provides detailed information identifying naturally processed FVIII peptides. The immunodominant T-cell epitopes that elicit immune responses in patients with HA must necessarily be contained within the repertoire of peptides presented on the relevant HLA Class II. Depending on their micro-environment and immunological context, regulatory and other subsets of CD4^+^ T cells may also recognize FVIII-derived peptides in the context of MHC class II. Retrospective studies based on chart reviews of HLA-typed patient populations have not revealed any clearly immunodominant HLA Class II alleles associated with inhibitor development (66–69).

In view of the intimate relationship between FVIII and VWF, it is not surprising that internalization of FVIII by antigen presenting cells or phagocytic cells involved in clearance can be influenced by VWF. Based on large genetic studies, single nucleotide polymorphisms in several proteins have been associated with circulating levels of VWF and FVIII (70). Polymorphic sites within genes linked to biosynthetic pathways such as *STX2* and *STXBP5* most likely affect circulating VWF levels through modulation of biosynthetic pathways in platelets and endothelial cells (71, 72). Conversely, polymorphic sites within surface receptors are expected to modulate circulating FVIII and VWF levels through their effect on the clearance of FVIII, VWF or the FVIII-VWF complex. Following up on the observations of the Cohorts for Heart and Aging Research in Genomic Epidemiology (CHARGE) Consortium has yielded unexpected insights into the clearance and immunogenicity of FVIII, pointing toward a prominent role for liver sinusoidal endothelial cells. The C-type lectin CLEC4M is a candidate receptor for regulating FVIII and VWF levels. *In vitro* expression studies revealed that CLEC4M can bind to both FVIII and VWF, positioning it as a potential regulator of FVIII clearance and immunogenicity (73, 74). Interestingly, CLEC4M is expressed exclusively in sinusoidal endothelial cells; infusion studies in CLEC4M deficient animals did not reveal major differences in levels of FVIII and VWF (73, 74). Another candidate receptor that arose from the CHARGE study is stabilin-2 (57). Stabilin-2 is a hyaluronan-binding receptor that is expressed primarily by liver sinusoidal endothelial cells. Like many endocytic receptors, stabilin-2 is a highly modular protein that is composed of a series of repeated domains. *In vitro* expression studies have identified both VWF and FVIII as potential ligands of stabilin-2 (45). Interestingly, infusion of FVIII-VWF complexes into stabilin-2 deficient mice resulted in a reduced immune response when compared to infusion of highly purified recombinant FVIII (45). Infusion of hyaluronic acid also resulted in a reduced immune response to FVIII. Altogether, these findings suggest that stabilin-2 can regulate the immunogenicity of FVIII, and that liver sinusoidal endothelial cells not only serve as a major, possibly exclusive site of FVIII synthesis (75, 76) but are also implicated in FVIII catabolism and immunogenicity.

## CD4^+^ T-Cell Response to FVIII

The involvement of CD4^+^ T cells in inhibitor development was first suggested by the clinical observation that HIV-positive HA patients, who tragically became infected through tainted blood products in the 1980s and were unfortunate enough to also have an inhibitor, showed a reduction in inhibitor titers as their T-cell counts declined (77). Qian *et al*. characterized FVIII-specific CD4^+^ T cells in a murine HA model (78) and demonstrated the critical role of T-cell dependent CD40-CD154 interactions in driving the antibody response (79). In a subsequent study, they identified an immunodominant CD4^+^ T-cell epitope recognized by these mice (80). The recent demonstration of expanded CD4^+^ T-follicular helper cells in spleens of FVIII-deficient mice with an inhibitor response has confirmed the expected essential role of this T-cell subset in providing B-cell help (43). Murine studies have also demonstrated an essential role for activated T cells in the memory B-cell response to FVIII, and the requirement for direct T-cell contact in order to re-stimulate these cells (81). Studies of patient blood samples have demonstrated CD4^+^ T-cell proliferation and cytokine secretion in response to FVIII protein and to synthetic peptides spanning the sequences of several FVIII domains (82–86). More recent investigations of the hemophilic immune response to FVIII have included isolation and characterization of human FVIII-specific T-cell clones and polyclonal lines (87–92), identifying immunodominant epitopes and phenotypes of minimally-expanded cells.

As mentioned earlier, autoimmune responses to self-FVIII can occur. Anti-FVIII antibodies isolated from acquired HA patient plasmas are class switched (23), indicating these inhibitors are flare-ups of a pre-existing but clinically insignificant autoimmune reaction. Interestingly, there is growing support for the notion that low-level T-cell auto-reactivity to endogenous FVIII may be a fairly common phenomenon in the healthy non-HA population. Several intriguing studies have indicated that many healthy individuals possess circulating CD4^+^ T cells that proliferate and secrete cytokines when stimulated with FVIII *in vitro* (93–95). Specificity of this T-cell response was further confirmed by a recent, elegant study in which FVIII-specific T-cell lines, which contained both naïve and memory subsets, were expanded from 16/16 non-HA blood donors (96). The calculated precursor frequency of FVIII-specific T cells was ~1.7 per million CD4^+^ T cells. These results indicate that thymic deletion of clones specific for the “self-protein” FVIII is incomplete, and that FVIII-specific memory T cells in non-HA individuals persist but do not expand [except in patients who develop neutralizing auto-antibodies to FVIII (4)]. A more recent study has identified FVIII epitopes recognized by CD4^+^ T cells from non-HA individuals using peptide ELISPOT assays and HLA Class II tetramers (97). Furthermore, peripheral blood mononuclear cells (PBMC) depleted of CD25^+^FoxP3^+^ cells showed enhanced proliferation compared to responses of non-depleted samples from non-HA subjects, suggesting that regulatory T cells (Tregs) play an important role in maintaining tolerance to endogenous FVIII under physiological conditions (93).

Thus, FVIII appears to be inherently more immunogenic than many other self-proteins (98, 99), and as-yet-undefined mechanisms maintain peripheral tolerance to self-FVIII in the vast majority of non-hemophilic individuals by preventing expansion of auto-reactive cells. Compare and contrast this situation with the development of anti-FVIII antibodies in severe HA, in which infused FVIII is a foreign protein: despite its inherent immunogenicity, ~¾ of these patients develop no neutralizing antibody (inhibitor) responses. Those who do develop an inhibitor may experience a transient neutralizing antibody response that either resolves spontaneously (100) or subsides following intensive FVIII infusions (ITI). Although low-titer anti-FVIII antibodies can often be detected in plasma/serum from these “tolerized” individuals, and FVIII-specific T-cell clones may still be isolated and expanded from their blood *in vitro* (88, 90), it is quite clear that peripheral tolerogenic mechanisms, which are still poorly defined, result in the desired clinical outcome of making FVIII replacement therapy possible by preventing or eliminating neutralizing FVIII-specific antibodies. Further studies of cellular responses to FVIII in HA patients with and without a high-titer inhibitor response, and in normal control blood donors, as well as animal model studies, are needed to clarify these mechanisms and potentially identify novel therapeutic targets.

Proliferation assays in which cells are stimulated with FVIII protein or peptides *ex vivo* (82–86, 95) have indicated that epitopes within multiple FVIII domains drive the anti-FVIII immune response. Analyses of FVIII-specific T-cell clones isolated using classic limiting dilution (87) or staining with peptide-loaded MHC Class II tetramers followed by cell sorting and expansion (88, 90–92) have unambiguously identified immunodominant T-cell epitopes in FVIII. Clones isolated from subjects with mild HA due to a missense mutation were, unsurprisingly, specific for epitopes corresponding to the wild-type FVIII sequence at the missense substitution site, as this is the only amino acid sequence in the infused FVIII that would be “foreign” to their immune system.

In another recent study, blood from a severe HA subject with a major *F8* gene deletion, who had failed ITI and had a persistent inhibitor, was analyzed by systematic epitope mapping using MHC Class II (HLA-DRA-DRB1^*^01:01 and HLA-DRA-DRB1^*^10:01) tetramers loaded with synthetic peptides spanning the FVIII A2, C1 and C2 domains (92). Given that FVIII is a large (~220 kDa) protein, one would expect to find a polyclonal T-cell response targeting multiple epitopes. Interestingly, and counter-intuitively, only one FVIII epitope produced tetramer staining above background levels. Furthermore, analysis of the T-cell receptor (TCR) repertoire of these FVIII-specific cells showed cells that stained most strongly for this tetramer (likely indicating high-avidity binding) had a very narrow, oligoclonal TCR repertoire. Together, these results are consistent with a role for clonal deletion and anergy, and perhaps regulatory T cells, as important components of the functional “peripheral tolerance” that most HA patients achieve, whereas clones that escape this elimination or down-regulation following exposure to infused FVIII (including high-intensity FVIII treatment as part of ITI therapy) can persist and continue to provide help to B cells leading to antibody secretion. It is worth mentioning that T-cell clones specific for this same HLA-DRA-DRB1^*^01:01-restricted epitope have also been identified in two mild HA subjects with the same allele (88–90). Together, these studies suggest that ITI deletes or anergizes the vast majority of FVIII-specific T-cell clones, and in cases where ITI fails, a polyclonal T-cell response has still been converted to a monoclonal or oligoclonal response. Further human studies are required to determine if this narrowing of the FVIII-specific T-cell repertoire is a general feature of ITI. If so, this may provide support for novel immune interventions, based on a limited number of HLA-restricted T-cell epitopes, to promote tolerance to the entire FVIII molecule in patients who have failed ITI (101).

**Figure 3:**
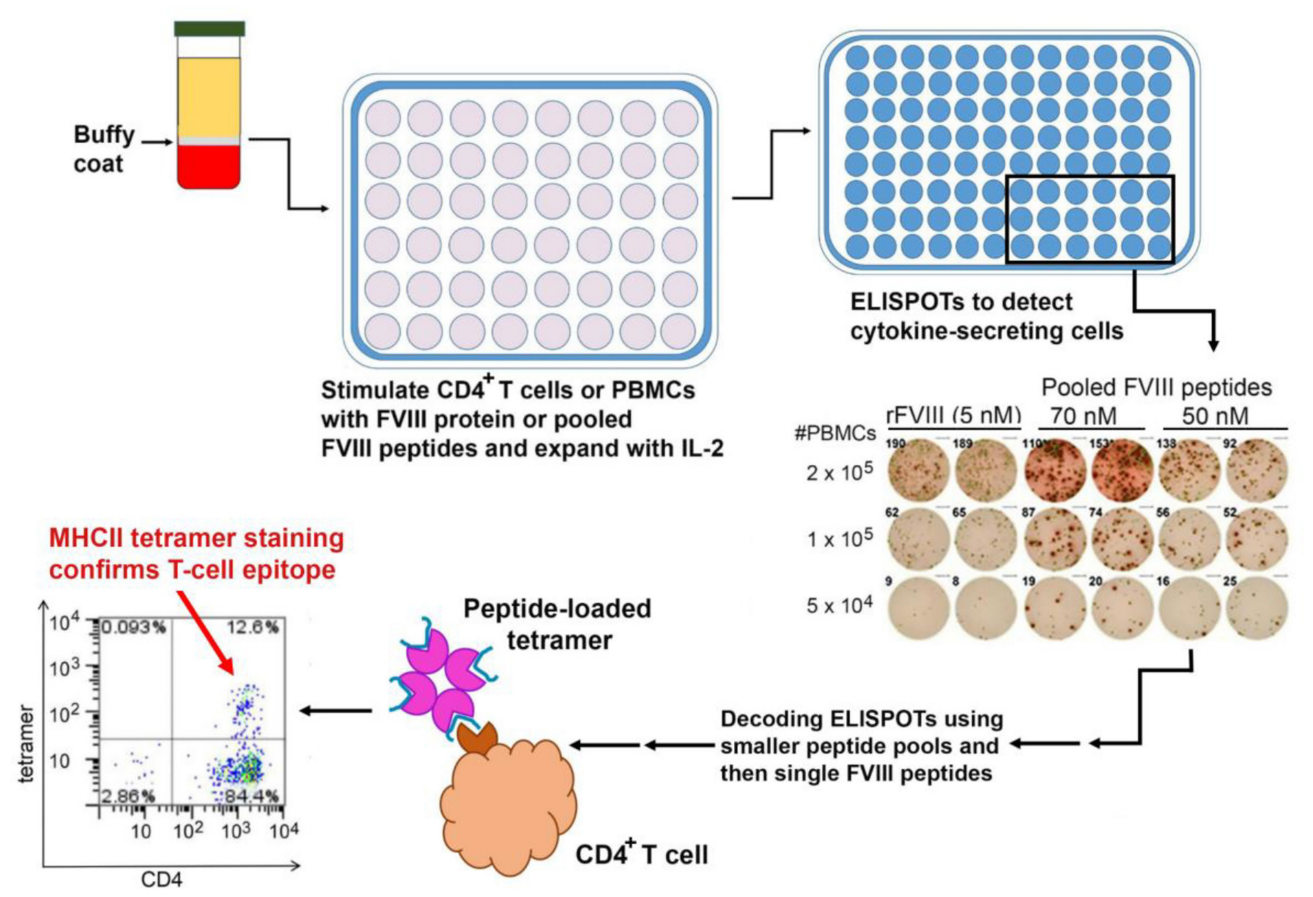
Mapping of HLA-restricted epitopes in FVIII recognized by CD4^+^ T cells from HA subjects. PBMCs are isolated from buffy coats of centrifuged blood and either used immediately or frozen. ELISPOT assays are carried out using either PBMCs or CD4^+^ T cells stimulated with FVIII protein or peptides, with unstimulated cells and tetanus-stimulated cells as negative and positive controls, respectively. Positive results from larger peptide pools are “decoded” by subsequent ELISPOT assays using smaller pools and then individual FVIII peptides as stimulants. Finally, ELISPOT results may be confirmed by staining CD4^+^ T cells using the appropriate peptide-loaded HLA Class II tetramers.

It is important to note that the study described above, which utilized HLA Class II tetramers, identified T-cell clones with high-avidity binding to a FVIII epitope. More recent studies employing ELISPOT assays to detect Th1 or Th2 cytokine secretion in response to FVIII peptides have identified responses to a larger epitope repertoire in a series of severe HA subjects (102) (Figure 3). This may well reflect lower-avidity peptide binding by these responsive cells, compared to the tetramer-positive cells, which is nevertheless physiologically relevant. Indeed, the respective roles of high- and low-avidity TCR binding interactions are of interest in studies of multiple allo-immune responses including allograft rejection and vaccine efficacy (103–105). As mentioned earlier, both antibody phenotyping and analysis of secreted cytokines have confirmed the involvement of both Th1 and Th2 subsets of CD4^+^ T-effectors in inhibitor development. Serial samples from one mild HA subject identified transient FVIII-specific (tetramer-positive) Th17/Th1 T cells 3–5 months following his initial inhibitor diagnosis, and parallel analysis of his FVIII-stimulated CD4^+^ T cells depleted of CD25^hi^ cells showed stronger tetramer staining, consistent with some suppression by CD25^hi^ Tregs (88). The ability of FoxP3^+^ Tregs to suppress inhibitor development in mice was demonstrated by Miao and colleagues, who found that transgenic HA mice that overexpressed FoxP3, unlike HA mice with unmodified FoxP3 expression, did not develop inhibitors following exposure to FVIII via plasmid-based gene therapy. Furthermore, adoptive transfer of CD4^+^CD25^+^FoxP3^+^ cells from the FVIII-exposed transgenic mice to the non-transgenic mice protected the recipient mice from developing high-titer inhibitors, and these Tregs also suppressed proliferation of FVIII-stimulated CD4^+^ T-effectors *in vitro* (106).

Further mechanistic studies in animal models and longitudinal studies of the anti-FVIII immune response in HA subjects are needed. The roles of FVIII-specific T-effector cells in patients with a persistent inhibitor also require further clarification; are these cells essential for maintenance of longstanding inhibitor responses, which are primarily driven by memory B cells?

## Roles of Complement and Oxidation in FVIII Immunogenicity

The study of the immunogenicity of therapeutic FVIII relies on the use of different *in vivo* and *in vitro* experimental models. The *in vitro* analysis of the endocytosis of FVIII by purified APCs presents the advantage of a controlled study system; it however fails to encompass the diversity of the populations of APCs that may co-exist at a given time point and in a given microenvironment *in vivo*, and fails to account for the varying flow and shear stress conditions that pre-exist in the different body compartments where therapeutic FVIII may be encountered. More importantly, the use in cell culture of serum-free medium or medium containing heat-inactivated serum leaves aside numerous clotting factors and other plasma proteins. These include molecules that have developed a specific relationship with FVIII, in particular VWF, which was shown to reduce FVIII uptake (59, 107), as well as molecules that are essential for innate and adaptive immunity, such as circulating immunoglobulins or complement.

The complement system is an integral part of the innate and adaptive host defense (108, 109). Activation of the complement cascade can occur through at least three pathways: (1) the classical pathway is activated when C1q binds to immune complexes; (2) the lectin pathway is elicited by the binding of mannose-binding lectin to mannose residues on pathogens; and (3) the alternative pathway is spontaneously and continuously activated at a low rate (i.e., spontaneous C3 tick-over) (110, 111). The inappropriate activation of complement is pathogenic and has been associated with autoimmune reactions (112).

Recent studies have investigated whether complement activation plays a role in the onset of the anti-FVIII immune response. The administration of humanized cobra venom factor (hCVF) to mice was followed by an exhaustion of C3 from the circulation without generation of the down-stream pro-inflammatory anaphylatoxin C5a (113). The treatment of FVIII-deficient mice with hCVF prior to replacement therapy resulted in a 4-fold reduction in the levels of neutralizing anti-FVIII IgG, as compared to PBS-treated mice (114). To gain molecular insight into the implication of complement C3 in FVIII immunogenicity, the endocytosis of FVIII by APCs was studied in the presence of heat inactivated (i.e., de-complemented) or non-heat-inactivated human AB serum. Heat inactivation of serum resulted in a 2-fold decrease in FVIII uptake by both immature monocyte-derived dendritic cells (MODCs) and conventional blood DCs (114). Decreased FVIII internalization resulted in a proportional decreased activation of FVIII-specific T cells. Interestingly, elevated levels of FVIII uptake (and T-cell activation) were restored when MODCs were co-incubated with the reconstituted C3 activation complex or with the C3 activation fragment C3b alone. In agreement with this, although the specific endocytic receptor has not yet been identified, FVIII and C3b co-localized at the cell surface. Of note, an engineered FVIII protein with three amino acid changes in its C1 domain, which showed reduced immunogenicity, was described a couple of years ago (52, 57). While this mutant FVIII was not endocytosed by MODCs *in vitro*, its uptake was rescued in the presence of complement activation (114). It is tempting to propose temporary C3 depletion with hCVF as a therapeutic strategy to prevent the development of anti-FVIII antibodies during initial FVIII infusions of naïve HA patients (Figure 2).

Bleeding is typically associated with hemolysis that leads to the release of hemoglobin and free heme, and with the release at the site of injury of several pro-inflammatory mediators, including reactive oxygen species (ROS) (115). ROS have been demonstrated to alter the structure, function and immunogenicity of various proteins (116–118). The production of rFVIII under oxygen-free conditions preserves its pro-coagulant activity (119), possibly owing to its sensitivity to oxidation (120). Controlling oxidation *in vivo* in FVIII-deficient mice using N-acetyl-cysteine (NAC) was demonstrated to significantly reduce the intensity of the immune response to therapeutic FVIII (121). Conversely, *ex vivo* oxidation of FVIII prior to administration to mice resulted in increased immunogenicity as compared with non-oxidized FVIII. The immunogenicity of the oxidized FVIII was, however, not reduced when mice were treated with NAC, suggesting that NAC does not merely affect the immune response but may act directly by preventing FVIII oxidation. An earlier study also identified FVIII as a heme-binding protein (122). The binding of heme to FVIII resulted in a partial loss of pro-coagulant activity, which was at least in part consecutive to a reduced capacity of FVIII to interact with activated FIX. The effects of oxidation of heme-bound FVIII on FVIII functions and immunogenicity remain to be investigated.

## Glycan Influences on FVIII Immunogenicity

Since the publication of the prospective randomized SIPPET study data, demonstrating a 1.87-fold increase in FVIII inhibitor incidence in previously untreated patients (PUPs) with the use of recombinant as opposed to plasma-derived FVIII, there has been a further search for factors that might provide a biological explanation for this difference (123). Furthermore, four independent cohort studies evaluating FVIII inhibitor incidence in PUPs have documented significant differences between a 2nd generation full-length rFVIII product and 3rd generation rFVIII concentrates, with the 2nd generation concentrate demonstrating a 1.6 to 2.8-fold increase in inhibitor incidence (124–128). Notably, the full-length 2nd generation product is expressed in baby hamster kidney (BHK) cells and the 3rd generation concentrates in Chinese hamster ovary (CHO) cells. While there are several possible explanations for the results of this series of epidemiological findings, a biologically plausible association relates to differences in the post-translational modifications, and specifically the glycosylation patterns found on various recombinant FVIII products produced in different cell types, and the plasma-derived protein derived from native human endothelial cells (54). A recent exploration of this glycosylation hypothesis has indeed provided supportive evidence for this proposal (129).

In this recent report, a range of methodologies have been used to document the glycan difference between rFVIII products and the subsequent effects on the FVIII immune response *in vitro* and in both “regular” (fully murine) *F8-*KO HA mice (*F8* exon-16 deletion) and in humanized hemophilic mice expressing a mutant human *F8* transgene product (with the hemophilia-causing mutation FVIII-Arg593Cys). Lectin binding and mass spectrometry analysis of the 2nd generation rFVIII and a full-length 3rd generation rFVIII concentrate showed a reduction in occupied N-linked glycosylation sites in the 2nd generation product and significant differences in the content of sialic acid and high mannose glycans. These structural differences were associated with increased immunogenicity in the mouse models of HA. In studies involving mice with the mutant human FVIII transgene (Arg593Cys), subcutaneous delivery of the BHK-expressed 2nd generation rFVIII resulted in a 94% incidence of FVIII inhibitors vs. 47% incidence following subcutaneous administration of the 3rd generation rFVIII. Anti-FVIII IgG titers were also significantly higher following exposure to the 2nd generation rFVIII.

In conclusion, these studies documented significant differences in the pattern of glycan occupancy and the types of glycans attached to rFVIII expressed in BHK vs. CHO cells. These differences were associated with variances in FVIII immunogenicity in mouse models of HA. These findings suggest that one of the factors influencing FVIII immunogenicity is the glycan profile, including both quantitative and qualitative details, at least in mice. They also suggest that there may be strategies involving glycan bioengineering that would be protective against FVIII immunogenicity. Preliminary results of an ongoing clinical trial testing a rFVIII product from a human cell line indicated that 17.6% of previously untreated patients developed a high-titer inhibitor (https://www.octapharma.com/news/corporate-news/2019/new-data-isth-2019/), suggesting that fine differences in glycosylation may not play a predominant role in FVIII immunogenicity in humans.

## Gut Microbiome Influences on FVIII Immunogenicity

Over the past decade, there has been rapidly growing evidence that alterations of the gut microbiome play a key role in regulating both local and systemic immune responses (130–132). The mechanisms underlying this influence are still being investigated, but they include molecular mimicry from gut microbial antigens and immunomodulatory effects of microbial-derived metabolites (133).

Whether the gut microbiome contributes to the risk profile for FVIII immunogenicity has yet to be investigated in detail, but preliminary results in a mouse model system suggest that this may indeed be the case. Furthermore, the timing of initial FVIII exposure in severe HA children, in the first 1–2 years of life, not only represents the peak period for FVIII inhibitor generation but also represents the period when inter-individual microbiome differences are at their most extreme, and when exposure to immunologic challenges such as vaccinations are also initiated (134).

**Figure 4:**
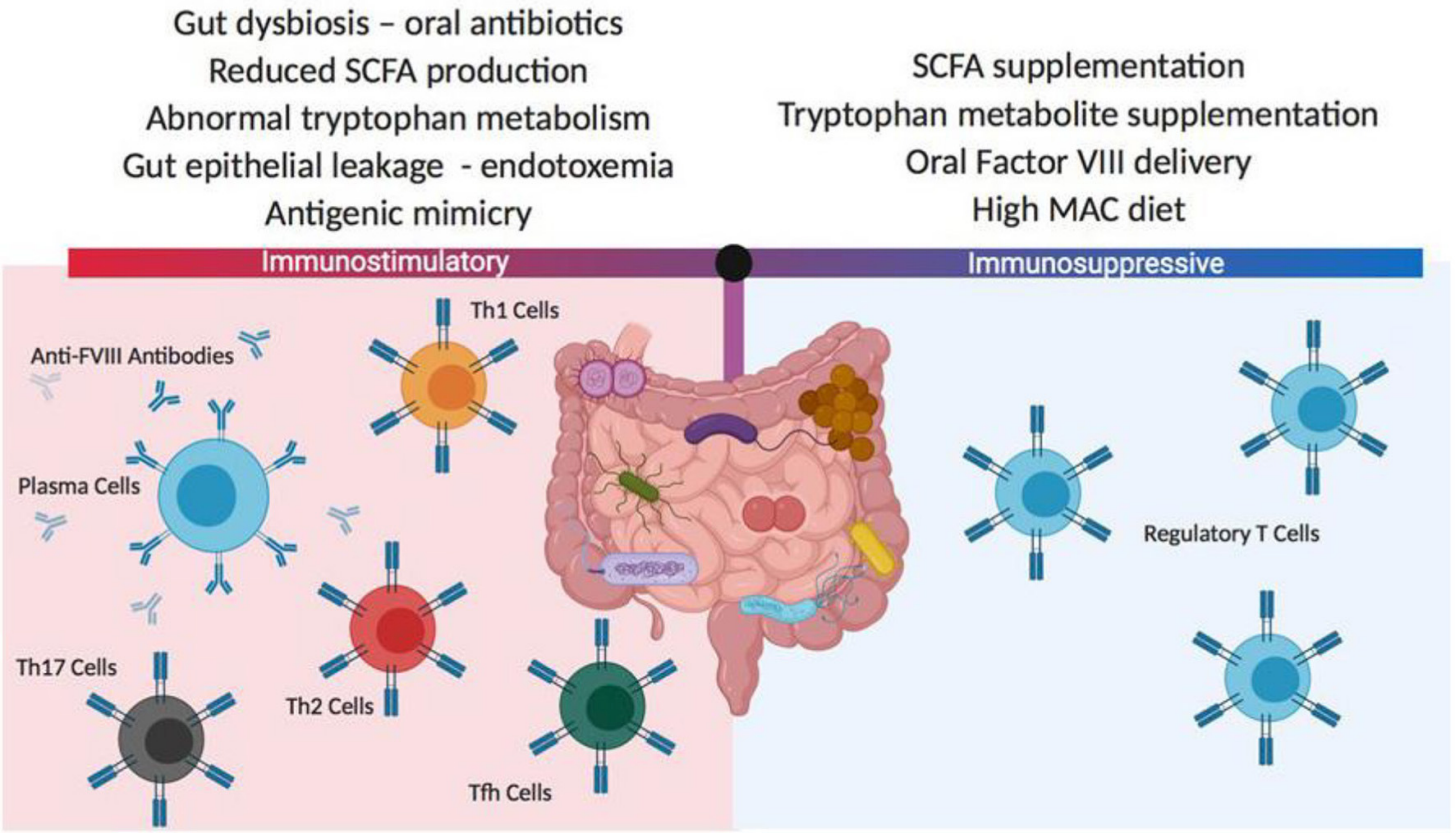
Potential gut microbiota influences on FVIII immunogenicity. The composition of the gut microbiome can have significant positive or negative effects on FVIII immunogenicity. These differences in the microbiome composition can derive from changes in diet, medications and coincident disease states. In most instances, the mechanisms responsible for changes to FVIII immune reactivity are not well-characterized but it is thought that antigenic mimicry and gut inflammation may play roles in initiating and boosting FVIII immunogenicity, while the delivery or generation of gut-derived immunomodulatory metabolites can mediate tolerogenic immune responses. SCFA, short chain fatty acids; MAC, microbiome accessible carbohydrate.

In a series of studies involving HA mice whose gut microbiome has been disrupted by oral administration of a broad-spectrum antibiotic, follow-up after repetitive FVIII challenge has been associated with significantly increased titers of anti-FVIII antibodies in the dysbiotic animals (135). Microbial analysis of the cecal contents in antibiotic-treated mice demonstrated significant reductions in Lactobacillus and Clostridia class immunomodulatory strains of bacteria. Detailed phenotyping of the antibiotic-treated and control mice at the time of initial FVIII exposure showed no differences in mesenteric lymph node and splenic regulatory T cell numbers, dendritic cell subsets and cytokine levels, but the cecal contents at this time demonstrated significantly reduced levels of the immune modulatory short chain fatty acids, acetate, propionate and butyrate. These initial observations provide a rationale for further evaluation of the gut microbiome as a contributing influence for FVIII immunogenicity. Whether interventions involving probiotic supplementation, specific immunomodulatory metabolite administration or microbiome- facilitated oral tolerance protocols can impact FVIII inhibitor development will require a considerable expansion of our current knowledge of this component of the body's immune system (136) (Figure 4).

## FVIII-Fc Fusion Proteins

It has long been appreciated that monomeric, heterologous immunoglobulin G (IgG), as well as model antigens coupled to Ig heavy chains (IgG H), are tolerogenic (137–140). The IgG heavy chain Fc region binds to Fc receptors, thereby inhibiting B-cell receptor signaling (141) to facilitate antigen uptake and tolerance. Indeed, early studies showed that coupling of haptens to IgG Fc led to tolerance, but coupling to F(ab')_2_ did not (142). The recent fusion of FVIII with a human IgG_1_ Fc has created a therapeutic FVIII with extended half-life due to recycling of the protein via the neonatal Fc receptor (FcRn) (143). Initial pre-clinical and clinical studies (144–147) have suggested that FVIII-Fc may also lower the incidence of inhibitor development and increase ITI success rates (148). A recent study based on retrospective chart review indicated that patients receiving FVIII-Fc for ITI tolerized faster than with standard ITI protocols utilizing non-Fc fusion products, and several patients receiving “rescue ITI,” i.e., who had failed an earlier ITI regimen, became tolerized during ITI with FVIII-Fc and were able to resume standard replacement therapy (149). Clinical trials to test the safety and efficacy of FVIII-Fc in previously untreated HA patients (NCT02234323), and in patients undergoing ITI (NCT03093480 and NCT03103542), are currently under way. In addition, a FVIII-Fc fused to the VWF D'-D3 fragment and to an XTEN polypeptide to further extend its half-life, and which can be delivered subcutaneously, is in phase 1 testing (NCT03205163).

In humans, maternal IgG are transferred to the fetus through the placenta during the third trimester of pregnancy. This transfer of IgG is not passive but involves binding of the Fc fragment of the IgG to the FcRn expressed by the syncytiotrophoblast (150). The binding between FcRn and IgG occurs after uptake of IgG into the acidic endosome and prevents routing of the internalized IgG to the lysosomes and degradation, thereby favoring transcytosis to the fetal circulation instead. The same occurs in mice, albeit in a different time frame, with maternal IgG being transferred to the fetus from day 15 of pregnancy onwards. Such a phenomenon was exploited in mice, wherein an Fc fusion version of β-glucuronidase injected into the pregnant animals was detected in the fetus (151). Incidentally, the third trimester of pregnancy in the human, and days 14–20 of pregnancy in the mouse, witness the development of the fetal immune system and establishment of tolerance to self (152). Administration to pregnant FVIII-deficient mice of Fc-fused A2 and C2 domains of FVIII is followed by the FcRn-dependent transfer of these molecules to the fetal compartment, followed by their transport by SIRPα^+^ dendritic cells to the thymus (153). The introduction of FVIII-A2-Fc and FVIII-C2-Fc during fetal life induced FVIII-specific regulatory T cells that were detected after birth, and that protected against alloimmunization to therapeutic FVIII later in life (154). Interestingly, preliminary data from the Lillicrap group showed that injection of high dose therapeutic FVIII-Fc (Eloctate) to pregnant mice allows detection of FVIII activity in the fetuses, which is not the case when recombinant FVIII alone is injected (155). Together, these data provide proof of concept for the antenatal induction of active and long-lasting immune tolerance to therapeutic FVIII, if FVIII is administered at the appropriate gestational stage. A similar approach was tested, with success, in animal models of Type I diabetes (156). In principle, this could be extended to prevent alloimmune responses to therapeutic agents used to treat other monogenic diseases too, such as hemophilia B (157) or Pompe disease (158).

Fc-FVIII fusions may be employed in novel cellular-based therapies designed to induce specific tolerance to the Fc-conjugated protein. Based on studies that demonstrated that B-cell presentation of antigens could be tolerogenic (159, 160), it was demonstrated that retroviral transduction of the FVIII A2 and C2 domains inserted in-frame at the N-terminus of isologous IgG H chains into B cells blocked and even reversed inhibitor formation (161). This system required MHC class II expression on the B cells, and led to the generation of regulatory T cells (Tregs) in multiple models of adverse immune responses (uveitis, EAE, diabetes, arthritis) in addition to HA (161–165).

## T-Cell Engineering for Tolerance

Polyclonal Tregs can be suppressive both *in vitro* and *in vivo*. However, because they contain multiple TCR from the entire repertoire, a potential drawback to their clinical application is that they could be non-specifically immunosuppressive. Indeed, there are anecdotal examples of viral re-activation in some clinical trials (166) and concerns about lowering immune barriers to cancer. Efforts to expand antigen-specific Tregs from polyclonal precursors are challenging; however, this has been achieved recently for HA mouse models (167, 168). The use of antigen-specific Tregs has also been significantly refined in an alternative approach using retroviral transduction to express a *single* TCR, derived from a HA subject and recognizing a well-defined HLA-restricted T-cell epitope in FVIII (89), on human Tregs. When this TCR was expressed in sorted human Tregs (CD25^+^, CD127^lo^, FoxP3^+^, Helios^+^), these engineered Tregs suppressed proliferation and cytokine secretion by FVIII-specific CD4^+^ T-effector clones. An important advance that made this tolerogenic approach possible was the development of methods to expand human Tregs *ex vivo* (169). Moreover, when the TCR-transduced Tregs were added to spleen cells from FVIII-immunized mice, antibody formation to FVIII was significantly inhibited, thereby demonstrating that bystander suppression to multiple epitopes in other domains of FVIII was occurring. This suppression was also demonstrated *in vivo*, despite the xenogeneic barrier and rejection of the human Tregs within 1–2 weeks. A fully murine system needs to be developed to test the durability of this tolerogenic effect.

These studies provided proof-of-principle for the utility of engineered Tregs, but the HLA restriction of TCRs would require development of patient-specific Treg lines, a formidable barrier. In order to develop antigen-specific Tregs that are not TCR/HLA-restricted, Tregs were next engineered to express a single chain Fv (isolated from a phage display library) recognizing the FVIII A2 domain (170). Tregs expressing this chimeric antigen receptor (CAR) were similarly effective as the TCR-engineered Tregs at suppressing FVIII antibody and inhibitor response *in vitro* and *in vivo*. Mechanistic studies suggested that contact between Tregs and T-effectors enhanced suppressive function driven by IL-2 (171), but the targets of the TCR- and scFv CAR Tregs might be different. The former act on antigen-presenting cells (expressing peptide:MHC) while the latter would be activated by conformational epitopes of the properly folded FVIII protein.

**Figure 5:**
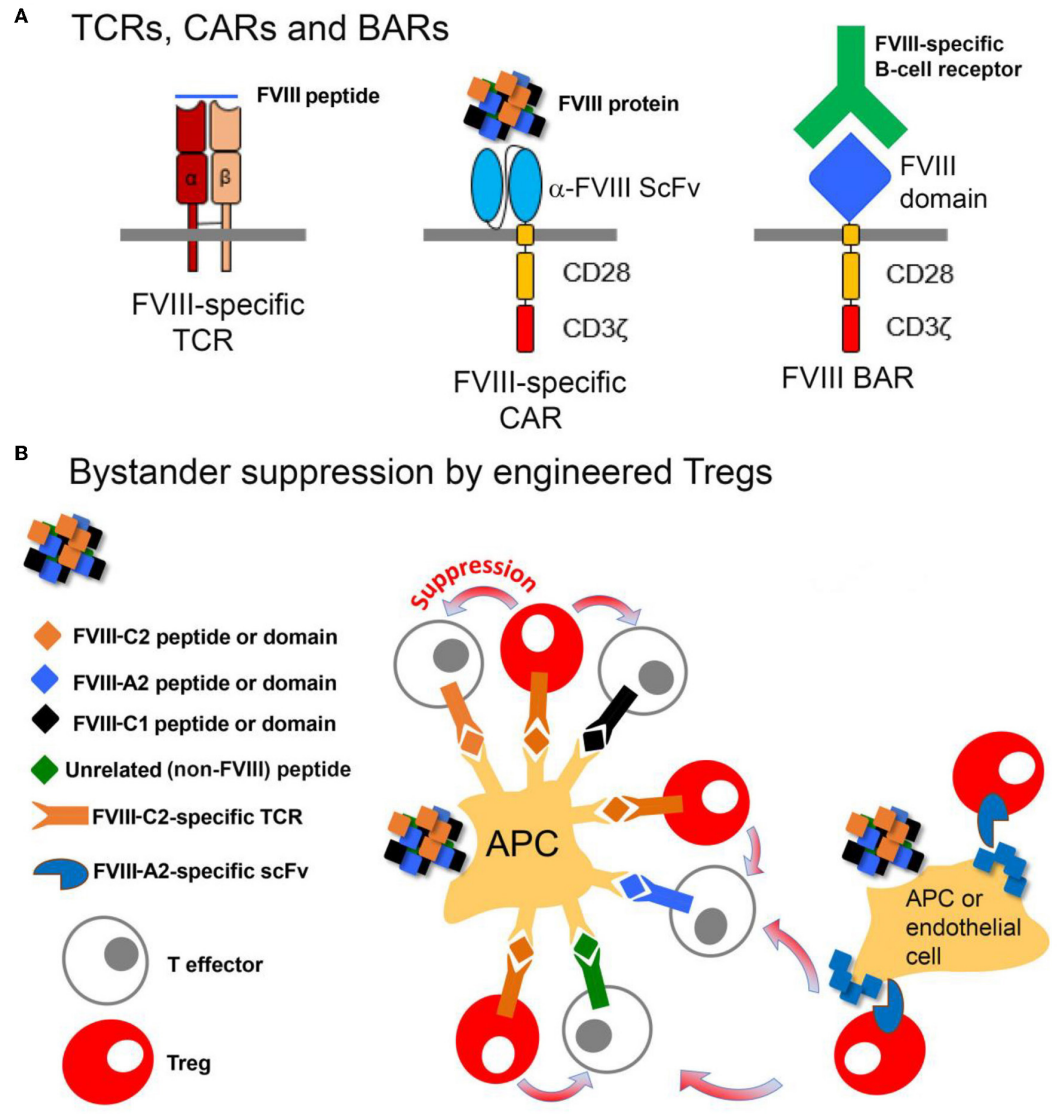
Design and function of Tregs engineered to express FVIII-specific TCRs, CARs or BARs. **(A)** The FVIII-TCR Tregs will recognize FVIII peptides with specific HLA-restricted T-cell epitopes. The FVIII-specific scFv Tregs will recognize FVIII domains, with no HLA restriction. The FVIII-BAR T cells will recognize B cells expressing FVIII-specific BCRs. FVIII-BAR Tregs are expected to down-regulate or prevent FVIII-specific B-cell activation, while FVIII-BAR CD8^+^ T cells should specifically kill only the FVIII-specific B cells. **(B)** Cartoon showing possible mechanisms by which engineered FVIII-specific Tregs may exert bystander suppression, i.e., create a tolerogenic environment that will suppress nearby T-effectors having specificity for multiple epitopes in FVIII.

As a further approach, FVIII domains have now been expressed on the surfaces of both Tregs and CD8^+^ cytotoxic cells, in order to directly target B cells. These engineered cells are referred to as B-cell Antigen Receptor, or “BAR” T cells, since the expressed domains would be recognized by FVIII-specific B-cell receptors (BCR). The Treg and CD8^+^ BARs suppressed and killed, respectively, FVIII-specific B cells, thereby blocking anti-FVIII antibody production (172, 173). Thus, these various engineered FVIII-specific Tregs (Figure 5) are demonstrably functional, with different targets and advantages. Ongoing studies are now testing their tolerogenic properties in the presence of high-titer FVIII inhibitors.

## Oral Tolerance

Delivery of antigens via the mucosal route has been known to be tolerogenic for decades, and this route of antigen exposure may lead to the induction of Tregs as well. One challenge for oral tolerance is that it requires large amounts of protein, the cost of which would be prohibitive with FVIII. Daniell and Herzog have overcome this challenge by designing a system in which encapsulated FVIII fused with cholera toxin subunit B (which enables transfer across the gut epithelium but is itself nontoxic) is expressed in lettuce leaves (174–176). The lettuce is then processed into powder in a GMP facility. When this FVIII-containing powder was fed to hemophilic mice and dogs, tolerance was induced in both prophylactic and therapeutic experiments (176). This approach appears to hold tremendous promise as a non-invasive method to promote tolerance to FVIII, even in advance of initial FVIII infusions (and hence before inhibitors could develop). Furthermore, the application of oral tolerance protocols in the context of immune modulatory gut microbial environments may provide additional benefits in the induction of tolerogenic responses to FVIII.

## Nanoparticles for Antigen-Specific Tolerance

In the last few years, nanoparticles (NP) that had been designed for drug delivery were also found to be efficient vehicles for tolerance induction. These self-assembling, biodegradable poly(lactide-co-glycolide) tolerogenic NPs (tNP) that contain the immune modulator, rapamycin, with or without protein or peptide antigens are capable of inducing durable antigen-specific tolerance that controls adaptive immune responses and can withstand multiple immunogenic challenges with antigen. Thus, Maldonado et al. utilized tNPs containing rapamycin given together with repeated doses of FVIII (177). This protocol blocked FVIII inhibitor production even with repeated FVIII challenge for over 200 days and led to Treg development. Moreover, this protocol was successful in previously immunized mice, albeit requiring additional (multiple) treatments with tNPs. Further commercial development of such tNPs (*SEL-212)* is proceeding to promote tolerance to uricase, an immunogenic agent used for gout therapy (https://www.selectabio.com/immtor/gouttherapy/phase2results/). There is considerable interest in testing nanoparticle + rapamycin therapy in other immunogenic therapies, and rapid advances in this field are expected over the next several years.

## Influence of New Hemophilia Therapies on Factor VIII Immunogenicity and Tolerance

Over the past decade, a range of innovative non-factor replacement approaches for the treatment of HA have been under development, and several of these therapies are now in the clinic (178). The influence of these treatments on the FVIII immune response and FVIII tolerance will be variable, with some approaches (e.g., rebalancing hemostasis strategies) having a less obvious potential impact on FVIII inhibitor development, while for other novel therapies there will be clear, either direct or indirect, consequences for the FVIII immune response. The most clearly influential of these treatments to date are the humanized bispecific antibody, emicizumab (“Hemlibra”), and FVIII gene therapy.

Emicizumab has demonstrated partial FVIII mimetic properties in a variety of *in vitro* tests (179) and has been shown in phase 3 studies of HA patients both with (7) and without (8) FVIII inhibitors using prophylactic emicizumab treatment to very significantly reduce annualized bleed rates. The bispecific antibody does not induce or exacerbate anti-FVIII antibody responses, and anti-emicizumab antibodies have been detected in <5% of treated patients to date. A major question that is currently unresolved is whether FVIII inhibitor patients being successfully treated with emicizumab prophylaxis should undergo ITI in an attempt to eliminate their neutralizing anti-FVIII antibody response. Initial clinical studies are now underway aimed at addressing this question, but basic immunologic principles would suggest that the efficacy of ITI should not be decreased in emicizumab users. On the contrary, their reduced inflammatory status accompanying restoration of hemostasis may even improve ITI success rates. Induction of tolerance to FVIII would enable the preferential use of FVIII to treat episodes of breakthrough bleeding in these patients and would avoid the less predictable outcomes obtained with bypass product treatment. Importantly, administration of FEIBA as a bypass agent when emicizumab is “on board” may be contra-indicated due to a possibly increased thrombotic risk when these therapeutics are combined (180, 181); ongoing and future monitoring of patients treated with FEIBA while still on emicizumab will generate sufficient data to properly evaluate this potential risk. The only other currently approved bypass agent is recombinant factor (F)VIIa, which is expensive and has a short half-life. However, recent studies have indicated that concomitant treatment with emicizumab + rFVIIa does not change the safety profile of rFVIIa. The relative effectiveness of FEIBA vs. rFVIIa in treating breakthrough bleeds for patients on emicizumab has not yet been established, due to the limited amount of time this bispecific antibody has been on the market. Anecdotally, some inhibitor patients seem to respond better to one bypass agent than to another during serious bleeds, so removing FEIBA from the available armamentarium could prove problematic in some cases. Therefore, a lack of tolerance to FVIII could constitute an additional clinical risk factor even for patients successfully receiving emicizumab prophylaxis, by narrowing the options to staunch potentially dangerous bleeding following accidents, trauma or surgery. We therefore suggest that ITI continues to be an entirely appropriate therapy for inhibitor patients, regardless of whether they are being successfully treated with emicizumab, as ongoing tolerance to FVIII provides a clear clinical benefit by increasing the available options to treat or prevent bleeds.

The other scenario that may require consideration concerns the use of emicizumab in infants (previously untreated or treated with any FVIII concentrate), where the practical convenience of infrequent sub-cutaneous administration will provide a significant advantage for care givers and patients. In this situation, FVIII might only be administered at the time of breakthrough bleeding, and thus there may be a potential for increasing the inhibitor risk through FVIII delivery only at times of “high immunologic danger” due to associated bleeding and inflammation. This concern could be mitigated by early low dose FVIII prophylaxis to induce peripheral tolerance to FVIII. Furthermore, it is highly likely that regular, intermittent re-exposures to FVIII will be required to maintain this tolerance in infants, children and adults who choose emicizumab (or other non-FVIII therapies) for prophylactic prevention of bleeding. The necessity of antigen persistence for immunologic unresponsiveness has been demonstrated in many scenarios and is an accepted principle of immunology (182). Further research will be required to determine the optimal FVIII doses and maximum intervals between these doses to maintain peripheral tolerance. This would preserve the option of future FVIII replacement therapy for HA patients who choose alternative, non-FVIII therapies.

After 25 years of pre-clinical development, FVIII gene therapy is now being successfully applied in late stage clinical trials (183). There is a strong likelihood that the first licensed FVIII gene therapy product will be available within the next 12 months. All clinical trials to date have involved adeno-associated viral vector (AAV) liver-directed gene transfer of a B-domain-deleted FVIII transgene construct. All enrolled patients (until now) have had no prior history of FVIII inhibitor development, and no FVIII inhibitors have been documented in their follow up post-vector administration (out to a maximum of 3 years). In previous pre-clinical animal studies, transient anti-FVIII immune responses have been seen in a few animals, but all have been eliminated with persistent expression of the FVIII transgene. Furthermore, in studies of HA dogs with pre-existing FVIII inhibitory antibodies, AAV-mediated delivery of a canine FVIII transgene has been successful in mediating tolerance to FVIII, and the dogs eventually demonstrated persistent, therapeutically relevant levels of FVIII expression (184). Based on these results, it is reasonable to propose that in patients with FVIII inhibitors where routine ITI has failed, a trial of liver-directed FVIII gene therapy might prove effective. Development of a formal clinical trial protocol for evaluation of this intervention would be essential. Theoretical advantages that a gene therapy strategy for ITI might have include the persistent as opposed to intermittent exposure to the FVIII antigen, the relatively stable concentration of circulating FVIII, and the fact that FVIII production is from the liver, a well-documented location for supporting tolerogenic immune responses (185). FVIII gene therapy has not been approved for pediatric patients, so inhibitor risk for previously untreated severe HA patients is unknown. Further animal model studies will allow improved estimates of this and other potential safety issues that must be addressed adequately before offering this experimental therapy to infants and children.

Another creative approach to re-balance hemostasis in the absence of functional FVIII is to inhibit specific anticoagulation pathways by targeting activated protein C, which is generated *in vivo* by the thrombin/thrombomodulin complex on the surface of endothelial cells. Activated protein C is a serine protease that cleaves and thereby inactivates the cofactors FVIIIa and factor Va. Its natural inhibitor is the serpin (serine protease inhibitor) protein C inhibitor, which despite its name is a promiscuous inhibitor of multiple serine proteases. James Huntington and colleagues recently engineered a novel serpin that is highly selective for activated protein C, and that corrected bleeding in HA mice (186, 187). Further testing to evaluate the safety of this approach is needed, but at present it offers the intriguing possibility of achieving hemostasis by administering this relatively long-lived protein therapeutic, even in the presence of neutralizing anti-FVIII antibodies. As with other non-FVIII therapies, no effect on FVIII immunogenicity would be expected, although co-administration with FVIII to either induce or maintain tolerance would allow patients to resume FVIII replacement therapy either prophylactically, or as needed to treat traumatic or breakthrough bleeds.

## Discussion

In this review we have presented an overview of recent insights into the immune response to FVIII and novel approaches to prevent, circumvent or reverse the development of neutralizing anti-FVIII antibodies. It is an exciting time to be working in this field, with some new therapies already in clinical trials and others showing promising results in animal models. Several practical challenges remain, many of them inherent to studies of rare clinical disorders such as HA. For example, given that the anti-FVIII immune response is most likely to develop during initial infusions of infants and toddlers, there is limited availability of the required blood volumes for mechanistic studies of inhibitor development in humans. Yet, these studies are vital to understand the basis of different clinical outcomes, especially given the many differences between the diverse human population vs. other species, notably inbred mice (188). The number of cells available from genetically well-characterized mice, and even large animal models, can also be a limiting factor for studies, e.g., when attempting to characterize splenic marginal zone cells, or vascular and sinusoidal endothelial cells. The emergence of increasingly sophisticated techniques to analyze small samples, e.g., by flow cytometry-based immunophenotyping (189, 190), mass cytometry (189, 191), TCRαβ repertoire profiling (192, 193), and improved “-omics” methodologies combined with bioinformatics (194, 195) holds tremendous promise for furthering research into antigen-specific immune responses, including the basis for FVIII immunogenicity and maintenance of peripheral tolerance to FVIII.

Increasing coordination between hemophilia care providers, funding agencies and, of course, the hemophilia community itself is enabling initiatives from large-scale registries and associated data sets (196) to the establishment of sample and data repositories (197). Studies utilizing these resources will not only further our understanding of FVIII immunogenicity and tolerance, but they are also likely to provide insights into immunogenicity of various other biotherapeutics, which comprise a growing proportion of biotechnology and pharmaceutical company portfolios.

The introduction of cryoprecipitate, over 50 years ago, to treat HA patients was followed by development of additional plasma-derived and recombinant FVIII concentrates with improved safety profiles, and more recent advances have included modest half-life extension through various modifications of the FVIII protein. Non-FVIII therapies are now beginning to transform the lives of inhibitor (and some non-inhibitor) patients, allowing them to re-balance hemostasis to avoid most breakthrough bleeds, even in the presence of neutralizing anti-FVIII antibodies. Possible longer-term risks associated with these alternative therapies are unknown, and there is still little experience with their use in settings of trauma or surgery. Also, many patients and families who have evaluated recently available novel therapies choose to begin or remain on FVIII therapy. Therefore, maintenance of tolerance to FVIII remains a high priority. It is hoped that further development of tolerogenic approaches such as those described in this review will lead to new therapies allowing HA patients to “tolerate,” and fully benefit from, FVIII replacement therapy.

## Author Contributions

All authors contributed equally to strategy discussions and to writing of this article.

### Conflict of Interest

KP, DS, and SL-D are inventors on FVIII-related patents. JV is an inventor on FVIII-related patents and has received research funding from Novo Nordisk and has acted as an advisor for Biotest. The remaining author declares that the research was conducted in the absence of any commercial or financial relationships that could be construed as a potential conflict of interest.
